# Trends in dietary salt sources in Japanese adults: data from the 2007–2019 National Health and Nutrition Survey

**DOI:** 10.1017/S0007114522001416

**Published:** 2023-02-28

**Authors:** Mai Matsumoto, Ryoko Tajima, Aya Fujiwara, Xiaoyi Yuan, Emiko Okada, Hidemi Takimoto

**Affiliations:** 1 Department of Nutritional Epidemiology and Shokuiku, National Institutes of Biomedical Innovation, Health, and Nutrition, 1-23-1 Toyama, Shinjuku-ku, Tokyo 162-8636, Japan; 2 Department of Social and Preventive Epidemiology, School of Public Health, University of Tokyo, Bunkyo-ku, Tokyo, Japan

**Keywords:** Dietary salt sources, Trend, Japan, NHNS

## Abstract

Identifying trends in dietary salt sources is essential for effectively reducing salt/Na intake. This study aimed to examine the trends in dietary salt sources among Japanese adults using the 2007–2019 National Health and Nutrition Survey data collected from 95 581 adults aged ≥ 20 years. Dietary intake was estimated using the 1-d household-based dietary record. Foods reported as potential sources of salt intake in Japan and other countries were categorised into twenty-one groups. Salt intake for each food group was adjusted using the density method based on the energy intake. Trends in dietary salt intake based on food sources by sex and age groups (20–39 years, 40–59 years and ≥ 60 years) were analysed using the Joinpoint Regression Program. Salt intake for each age group in both men and women decreased from 2007 (5·3 g/1000 kcal–6·4 g/1000 kcal) to 2019 (4·9 g/1000 kcal–5·6 g/1000 kcal). The major dietary source of salt continued to be seasonings such as soya sauce and soyabean paste (approximately 70 %). Salt intake from seasonings decreased over time in adults aged ≥ 40 years but did not change in those aged 20–39 years. Additionally, a decreasing salt intake from unprocessed fish and shellfish and an increasing salt intake from unprocessed meat were observed across all age categories for both sexes. This study demonstrated that a strategy targeting different age groups may be needed to reduce salt consumption from seasonings among the Japanese population. Further studies on salt content in seasonings and continued monitoring of trends in dietary salt sources are required.

Excessive salt/Na intake is associated with lifestyle-related diseases, such as hypertension, CVD, stroke, kidney disease and stomach cancer^(1–4)^. Therefore, the WHO recommends a salt intake of < 5 g/d^(5)^. Additionally, a global voluntary target of 30 % relative reduction in the mean population intake of salt/Na by 2025 has been set by WHO member states^(6)^. However, despite the overall progress in salt/Na intake reduction efforts, many countries continue to consume more salt than is recommended by WHO, which remains a significant global health issue^(7–9)^. In particular, the salt intake in Asian regions, including Japan, is higher than that in other regions^(7,10,11)^. Although the Japanese salt intake has been decreasing, as of 2019, the daily salt/Na intake was approximately 10 g^(12)^, which is more than double the WHO recommendations. Therefore, additional efforts to reduce the salt/Na intake of the Japanese population are required.

To effectively implement population-based interventions to reduce salt/Na intake, dietary salt/Na sources, particularly trends in their consumption, must be identified^(13)^. Several studies have examined the sources of salt and Na intake. For example, grains, including breads and cereals, are the major sources of Na intake in the USA, the UK and Australia^(11,14)^. Meanwhile, in Brazil and China, salt added during cooking and at the table is the primary intake source of Na^(11,15)^. During 1996–1999, most dietary Na in Japan was from seasonings (especially soya sauce), soups (especially miso soup), fish and pickled vegetables^(11)^. Since 2010, cross-sectional studies have also reported that the main sources of dietary Na intake among the Japanese are seasonings (including soya sauce and soyabean paste), fish, noodles and bread^(16,17)^, which may indicate that food sources of salt/Na have changed over time.

Only a few studies have examined trends in the sources of salt/Na intake in the world. For example, data from the 1999–2010 National Health and Nutrition Examination Survey (NHANES) reported a decrease in Na intake from bread and fish and an increase in Na intake from salad dressings^(18)^. On the other hand, to date, there are no studies examining the time trends in dietary salt from food sources in East Asia, including Japan, where dietary salt sources differ from those in Western countries due to different food environments and dietary patterns^(8)^. It is suggested that strategies to decrease salt intake in China, a part of East Asia, should be different from those implemented in Western countries^(7,19)^, because seasonings are the main dietary source of salt intake in China, similar to Japan but different from Western countries^(11,14)^. Thus, it is important to evaluate the trend in dietary salt sources to develop effective strategies and guidelines to decrease salt intake in East Asian countries. Therefore, this study aimed to examine the trends in dietary salt sources in Japan using data from the 2007–2019 National Health and Nutrition Survey (NHNS).

## Methods

### Study design and data

The NHNS is a nationally representative cross-sectional annual survey conducted by local public health centres under the supervision of the Ministry of Health, Labour and Welfare, Japan^(20)^. This study examined data from the 2007–2019 NHNS, for which detailed dietary records were maintained. Details of the survey design have been described elsewhere^(20,21)^. Briefly, the surveys were conducted in November, except for the 2012 and 2016 surveys, which were conducted from 25 October to 7 December and 1 October to 30 November, respectively. The NHNS consisted of a physical examination, a dietary survey and a lifestyle questionnaire. Participants included households with family members (aged ≥ 1 year as of 1 November on the survey year) residing in 300-unit blocks (approximately 5700 households and 15 000 individuals) that were randomly selected from the unit blocks of the Comprehensive Survey of Living Conditions each year, except for 2012 and 2016, when an expanded survey was conducted. In 2012 and 2016, 475 (of approximately one million) census units were randomly stratified using a single-stage cluster sample design, and all household members in the selected area (approximately 23 750 households and 61 000 individuals) were invited to participate. The following areas were excluded from the analysis in specific years as noted because the survey was not conducted in these areas owing to natural disasters: Iwate, Miyagi and Fukushima prefectures because of the Great East Japan Earthquake in 2011^(22)^; Kumamoto prefecture by Kumamoto earthquake and Typhoon No. 10 in 2016^(23)^; Tottori prefecture by Tottori Prefecture earthquake in 2016^(23)^; and some areas of Nagano prefecture by Typhoon Hagibis in 2019^(12)^. Household response rates for each year in the NHNS ranged from 44·4 % in 2016 to 68·6 % in 2010.

This survey was conducted according to the guidelines laid down in the Declaration of Helsinki; all participants gave informed consent to the local government based on the Health Promotion Act^(24)^. Based on official application procedures under Article 33 of the Statistics Act, we obtained approval from the Ministry of Health, Labour and Welfare, Japan, to use individual-level data from the NHNS for this study. In accordance with the Ethical Guidelines of Epidemiological Research, approval from the Institutional Review Board was not required.

### Study participants

This study included 120 639 adults aged ≥ 20 years who participated in the NHNS dietary assessment survey. We excluded lactating or pregnant women who may have changed their usual dietary habits (*n* 1517)^(25)^ and individuals with missing data, such as body height and/or body weight (*n* 23 541). Thus, the data of 95 581 Japanese adults aged ≥ 20 years (43 129 men and 52 452 women) were examined in this study. The participants were classified into three groups according to age (20–39 years, 40–59 years and ≥ 60 years) as salt intake^(15,20)^, and its food sources^(17)^ could vary by age.

### Dietary assessment

Dietary intake data were collected using a 1-d semi-weighed household dietary record, excluding Sundays and public holidays^(20)^. Trained fieldworkers (mainly registered dieticians) visited each household to explain how to complete the dietary record. The main recordkeepers in the household (members who are usually responsible for preparing meals) weighed all foods and beverages consumed by each household member, as well as the food waste and leftovers, and noted their names and weights on recording forms. Additionally, the main recordkeepers recorded the approximate proportions of food consumed by each household member when members shared foods from the same dish to estimate individual intake. If weighing was not possible because the meal was consumed away from home, the main recordkeepers asked the family member regarding the portion size or quantity of foods consumed and details on any leftovers and recorded them.

Trained fieldworkers visited each household after a dietary record day and checked for missing information and errors. Regarding the foods and beverages that were not measured, the trained fieldworkers converted the estimates of portion sizes or quantity of foods into food weights and coded each food item according to the NHNS food number lists based on the Standard Tables of Food Composition in Japan^(26–28)^ to calculate the intake of energy and nutrients. The trained fieldworkers finally inputted collected dietary intake data using a software specifically developed for the NHNS, and the data were compiled by trained investigators at the central office to create an overall dietary dataset^(20)^.

Energy and nutrients were calculated based on the Standard Tables of Food Composition in Japan 2005 edition^(26)^ (data from 2007 to 2010), 2010 edition^(27)^ (data from 2011 to 2017) and 2015 edition^(28)^ (data in 2018 and 2019). The revision of the Standard Tables of Food Composition in Japan from the 2010 to the 2015 edition resulted in an increase in the number of food items categorised in the seasoning group (e.g. sweet soya sauce, ponzu (soya sauce with citrus juice), dressings and cooking sauces such as demi-glace sauce and nabe sauce (seasoned soup stock))^(27,28)^. Therefore, until the release of the 2017 data, these foods were previously replaced by foods such as soya sauce and soyabean paste when coding foods to estimate nutrient intake.

Based on previous studies in Japan and other countries^(11,14,16,17)^, we classified food sources of Na/salt into twenty-one groups as follows (each food group is defined in online Supplementary Table 1): grains, bread, noodles, instant noodles, vegetables, pickled vegetables, unprocessed fish and shellfish, dried fish and shellfish, processed fish and shellfish, unprocessed meats, processed meats, seasonings, kitchen or table salt, soya sauce, soyabean paste, sauce, soup stock, dressing, mayonnaise, roux, and other seasonings. Salt intake was estimated from the twenty-one food groups. Additionally, salt intake from each food group and each food group intake were adjusted using the density method based on the energy intake (i.e. amount of food group per 1000 kcal).

### Other variables

Body height (to the nearest 0·1 cm) and weight (to the nearest 0·1 kg) were measured for approximately 90 % of the participants by trained field workers in accordance with standard operating procedures^(22)^. For other participants, height and weight were measured by other household members at home or were self-reported. BMI was calculated by dividing weight (kg) with height (m) squared.

### Statistical analysis

Mean values and standard errors for age, BMI, energy intake and salt intake from the twenty-one food groups were calculated according to sex, age groups and survey years. For 2012 and 2016, the sampling weight of participants in each prefecture was calculated by dividing the total number of households during the past 3 years in each prefecture by that in 2012 and 2016, respectively, because the cluster sampling method used was different from that used in other years^(23,29)^. Although the NHNS is a household survey, households were not considered in the clusters. This is because we analysed the data by sex and age groups, and the proportion of multiple participants from the same household classified into the same sex and age group was approximately 1 % in the present study.

Trend analyses were conducted using the Joinpoint Regression Program (Joinpoint Regression software, version 4.9.0.0; National Cancer Institute, USA [https://www.surveillance.cancer.gov/joinpoint]). The Joinpoint regression analysis uses statistical criteria to determine the minimum number of linear segments required to describe a trend and the annual percentage change (APC) in each segment. When a change in trend is observed, the tables show the beginning year of the same trend as the lower point and the year at the end year of the trend as the upper point. The Monte Carlo permutation method was used to determine whether a change in the trend was statistically significant^(30)^. Other calculation and statistical analyses were conducted using the SAS statistical software (version 9.4; SAS Institute Inc.). Statistical significance was set at two-tailed *P* < 0·05.

## Results

The basic characteristics of the study participants are listed in Table 1 and 2. The mean age of both male and female participants aged 20–39 years did not change; however, there was a slight decrease in that aged 40–59 years and a slight increase in that aged ≥ 60 years. The mean BMI increased in men except for those aged 20–39 years. In contrast, there was no change in the mean BMI across all age categories for women.

**Table 1.** Characteristics of 43 129 Japanese men in the NHNS 2007–2019 (Mean values and standard deviations)

		2007		2008		2009		2010		2011		2012		2013		2014		2015		2016		2017		2018		2019		APC (%)	*P* *
*n*	20–39 years	643		608		609		549		533		1851		534		432		402		1318		381		391		267			
	40–59 years	927		885		928		791		742		2573		737		723		659		2210		632		701		507			
	≥ 60 year	1187		1361		1227		1196		1155		4508		1270		1329		1173		3989		1103		1107		991			
		Mean	sd	Mean	sd	Mean	sd	Mean	sd	Mean	sd	Mean	sd	Mean	sd	Mean	sd	Mean	sd	Mean	sd	Mean	sd	Mean	sd	Mean	sd		
Age (year)	20–39 years	31·3	5·6	30·9	5·9	31·0	5·6	31·5	5·7	31·5	5·8	31·2	5·8	30·5	6·1	31·2	5·8	31·4	5·6	31·3	5·7	30·9	5·8	30·9	5·8	30·3	6·0	–0·1	0·418
	40–59 years	50·1	6·0	50·5	5·9	50·0	6·0	49·8	6·0	50·0	6·0	49·7	6·0	49·4	6·0	49·8	6·0	49·5	5·9	49·5	5·9	49·3	5·8	49·3	5·7	49·3	5·7	–0·2	< 0·001
	≥ 60 years	70·5	7·3	70·7	7·4	70·7	7·5	70·6	7·5	71·1	7·5	70·8	7·5	71·3	7·3	71·2	7·4	71·4	7·5	71·7	7·6	72·0	7·5	72·0	7·5	71·9	7·3	0·2	< 0·001
BMI (kg/m^2^)	20–39 years	23·3	4·0	23·0	3·6	23·4	4·1	23·2	3·6	23·5	4·0	23·3	3·9	23·1	4·0	23·1	3·9	23·7	4·2	23·4	3·9	23·8	4·1	23·2	3·7	23·5	3·9	0·1	0·110
	40–59 years	23·8	3·3	24·0	3·3	23·9	3·3	24·2	3·4	24·0	3·5	24·1	3·3	23·9	3·3	24·0	3·5	24·0	3·6	24·3	3·8	24·1	3·5	24·2	3·5	24·7	3·9	0·1	0·006
	≥ 60 years	23·5	3·3	23·4	3·0	23·4	3·1	23·4	3·1	23·5	3·1	23·5	3·0	23·4	3·0	23·4	3·1	23·5	3·1	23·7	3·1	23·7	3·0	23·8	3·1	23·7	3·4	0·1	0·005
Energy intake (kcal/d)	20–39 years	2227	630	2138	622	2182	681	2137	652	2152	677	2171	635	2162	590	2136	695	2212	646	2134	639	2133	637	2238	641	2158	639	–0·1	0·556
	40–59 years	2203	613	2170	578	2198	579	2183	595	2130	556	2185	575	2139	563	2179	586	2195	597	2146	577	2170	631	2200	630	2208	621	–0·1	0·536
	≥ 60 years	2106	588	2093	548	2043	543	2051	542	2052	537	2099	542	2126	540	2118	545	2098	542	2096	528	2122	528	2128	515	2134	528	0·1	–

NHNS, National Health and Nutrition Survey; APC, annual percentage change; SD, standard deviations.

* The *P*-value was analysed by the Joinpoint regression analysis.

**Fig. 1.**
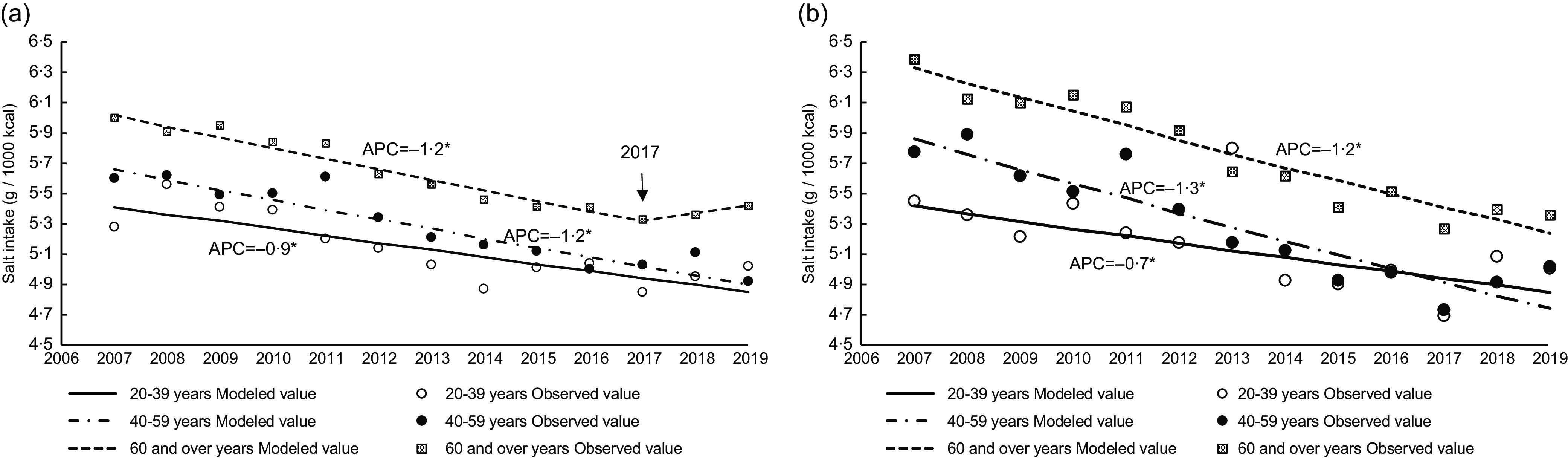
Trends in salt intake according to age group in Japanese men (a) and women (b) based on 2007–2019 NHNS data. Trends in salt intake were analysed by the Joinpoint regression analysis (*P* < 0·05). APC, annual percentage change; NHNS, National Health and Nutrition Survey.

The trends in salt intake according to age between 2007 and 2019 are shown in Fig. 1(a) and (b). Salt intake for each age group in both men and women, except for men aged ≥ 60 years, decreased from 2007 (5·3 g/1000 kcal–6·4 g/1000 kcal) to 2019 (4·9 g/1000 kcal–5·6 g/1000 kcal) with an APC of 0·7–1·3 %. In men aged ≥ 60 years, salt intake had a significant annual decrease of 1·2 % from 2007 to 2017. Tables 3 and 4 show the trend in salt intake from food groups except for seasonings from 2007 to 2019 in men and women, respectively. Salt intake from bread in adults aged ≥ 60 years and from noodles in women aged ≥ 60 years increased from 2007 to 2019, respectively. A decreasing salt intake from vegetables, pickled vegetables (except for men aged 20–39 years), and unprocessed fish and shellfish was observed across all age categories in both men and women. Additionally, salt intake from dried fish and shellfish in women aged 40–59 and ≥ 60 years, and from processed fish and shellfish in both men and women aged 40–59 years, decreased over time. Salt intake from meat in all age categories for both men and women showed significant increases; a sharp increase in men and women aged 40–59 years over the last 3 years was observed with APC values of 23·7 % and 28·9 %, respectively. Salt intake from processed meat increased only among men aged ≥ 60 years.

**Table 2.** Characteristics of 52 452 Japanese women in the NHNS 2007–2019 (Mean values and standard deviations)

		2007		2008		2009		2010		2011		2012		2013		2014		2015		2016		2017		2018		2019		APC (%)	*P* *
*n*	20–39 years	764		701		692		629		602		2012		539		477		409		1398		325		384		288			
	40–59 years	1114		1110		1094		996		893		3464		889		904		931		2807		793		809		675			
	≥ 60 year	1439		1673		1604		1509		1451		5709		1545		1545		1400		5066		1335		1329		1148			
		Mean	sd	Mean	sd	Mean	sd	Mean	sd	Mean	sd	Mean	sd	Mean	sd	Mean	sd	Mean	sd	Mean	sd	Mean	sd	Mean	sd	Mean	sd		
Age (year)	20–39 years	31·5	5·7	31·0	5·7	31·2	5·9	31·4	5·7	31·3	5·9	31·7	5·7	31·3	6·0	30·8	6·0	30·8	5·9	31·5	5·8	30·9	5·8	31·1	5·7	30·9	5·7	–0·1	0·545
	40–59 years	50·3	6·1	50·8	6·0	50·0	5·9	50·2	6·0	49·8	5·9	49·9	6·0	49·4	5·9	49·7	6·0	49·3	5·9	49·8	5·8	49·5	5·8	50·1	5·7	49·9	5·6	–0·1	0·033
	≥ 60 years	70·9	7·8	71·4	8·0	71·4	8·1	71·3	7·8	71·8	8·2	71·3	8·0	71·5	7·7	71·5	8·0	71·6	7·9	72·1	8·0	72·3	7·7	72·3	8·1	72·2	7·7	0·1	< 0·001
BMI (kg/m^2^)	20–39 years	20·9	3·1	21·1	3·6	21·1	3·7	21·2	3·3	21·2	3·3	21·3	3·6	21·2	3·6	21·7	3·9	20·9	3·0	21·3	3·7	21·3	3·7	21·2	3·4	21·5	3·4	0·1	0·054
	40–59 years	22·5	3·5	22·4	3·3	22·5	3·8	22·3	3·6	22·5	3·5	22·4	3·6	22·3	3·9	22·4	3·6	22·4	4·0	22·5	3·8	22·5	3·8	22·3	3·6	22·3	3·8	0	0·520
	≥ 60 years	23·1	3·7	23·0	3·6	23·0	3·5	23·1	3·5	22·9	3·6	23·0	3·5	22·8	3·6	22·8	3·6	22·8	3·4	22·9	3·6	23·0	3·6	23·0	3·7	22·9	3·6	0	0·173
Energy intake (kcal/d)	20–39 years	1698	461	1658	460	1684	447	1644	475	1622	465	1652	459	1648	486	1630	457	1648	493	1632	450	1704	552	1684	471	1625	447	–0·2	0·137
	40–59 years	1759	436	1758	471	1741	426	1728	443	1717	446	1737	444	1687	425	1709	450	1729	466	1699	445	1738	461	1739	437	1720	451	–0·2	0·042
	≥ 60 years	1700	452	1696	438	1670	432	1682	426	1659	428	1696	441	1705	451	1671	409	1716	448	1717	439	1747	447	1752	447	1766	531	0·3	0·004

NHNS, National Health and Nutrition Survey; APC, annual percentage change; SD, standard deviations.

* The *P*-value was analysed by the Joinpoint regression analysis.

**Table 3.** Trends in salt intake from food groups except for seasonings according to age group in Japanese men based on 2007–2019 NHNS data (Mean values and standard error)

		2007		2008		2009		2010		2011		2012		2013		2014		2015		2016		2017		2018		2019		Lower end point	Upper end point	APC (%)	*P* *
*n*	20–39 years	643		608		609		549		533		1851		534		432		402		1318		381		391		267					
	40–59 years	927		885		928		791		742		2573		737		723		659		2210		632		701		507					
	≥ 60 years	1187		1361		1227		1196		1155		4508		1270		1329		1173		3989		1103		1107		991					
g/1000 kcal		Mean	se	Mean	se	Mean	se	Mean	se	Mean	se	Mean	se	Mean	se	Mean	se	Mean	se	Mean	se	Mean	se	Mean	se	Mean	se				
Grains	20–39 years	0·52	0·04	0·55	0·04	0·59	0·04	0·56	0·04	0·50	0·03	0·53	0·02	0·57	0·04	0·57	0·04	0·53	0·04	0·51	0·02	0·53	0·04	0·58	0·04	0·56	0·05			–0·1	0·829
	40–59 years	0·44	0·02	0·46	0·03	0·48	0·03	0·43	0·02	0·46	0·03	0·47	0·01	0·45	0·02	0·45	0·02	0·46	0·02	0·47	0·02	0·48	0·03	0·56	0·03	0·54	0·03			1·3	0·011
	≥ 60 years	0·42	0·02	0·45	0·02	0·44	0·02	0·47	0·02	0·48	0·02	0·42	0·01	0·47	0·02	0·48	0·02	0·48	0·02	0·45	0·01	0·51	0·02	0·46	0·02	0·49	0·02			0·9	0·087
Bread	20–39 years	0·16	0·01	0·16	0·01	0·17	0·01	0·20	0·01	0·17	0·01	0·17	0·01	0·19	0·01	0·17	0·01	0·18	0·01	0·17	0·01	0·20	0·02	0·14	0·01	0·17	0·02			–0·2	0·838
	40–59 years	0·18	0·01	0·15	0·01	0·17	0·01	0·18	0·01	0·18	0·01	0·18	0·01	0·19	0·01	0·19	0·01	0·20	0·01	0·18	0·01	0·19	0·01	0·17	0·01	0·18	0·01			0·7	0·204
	≥ 60 years	0·20	0·01	0·19	0·01	0·19	0·01	0·21	0·01	0·21	0·01	0·19	0	0·23	0·01	0·23	0·01	0·24	0·01	0·20	0	0·24	0·01	0·22	0·01	0·23	0·01			1·3	< 0·001
Noodles	20–39 years	0·10	0·01	0·08	0·02	0·10	0·01	0·11	0·01	0·10	0·01	0·11	0	0·10	0·01	0·11	0·01	0·10	0·01	0·11	0·01	0·11	0·01	0·18	0·02	0·14	0·02	2007	2009	0·0	–
																												2009	2012	0·0	–
																												2012	2019	2·4	0·474
	40–59 years	0·09	0·01	0·07	0·01	0·09	0·01	0·08	0·01	0·09	0·01	0·09	0	0·10	0·01	0·08	0·01	0·09	0·01	0·08	0	0·08	0·01	0·13	0·01	0·12	0·01			–2·9	< 0·001
	≥ 60 years	0·19	0·01	0·18	0·01	0·20	0·01	0·21	0·01	0·21	0·01	0·18	0	0·23	0·01	0·22	0·01	0·23	0·01	0·20	0·01	0·24	0·01	0·22	0·01	0·23	0·01	2007	2012	0·0	–
																												2012	2015	8·9	0·345
																												2015	2019	–0·8	0·777
Instant noodles	20–39 years	0·20	0·03	0·24	0·04	0·25	0·04	0·21	0·03	0·17	0·03	0·21	0·02	0·24	0·03	0·24	0·04	0·19	0·03	0·18	0·02	0·17	0·03	0·19	0·03	0·17	0·04			–2·0	0·059
	40–59 years	0·12	0·02	0·12	0·02	0·17	0·02	0·12	0·02	0·14	0·02	0·16	0·01	0·12	0·02	0·12	0·02	0·13	0·02	0·16	0·01	0·17	0·03	0·19	0·03	0·19	0·03			–0·1	0·945
	≥ 60 years	0·09	0·02	0·12	0·02	0·13	0·02	0·15	0·02	0·14	0·02	0·12	0·01	0·12	0·02	0·12	0·02	0·12	0·02	0·12	0·01	0·14	0·02	0·10	0·01	0·12	0·02			–1·1	0·282
Vegetables	20–39 years	0·27	0·02	0·24	0·02	0·27	0·02	0·27	0·02	0·23	0·02	0·21	0·01	0·17	0·01	0·18	0·02	0·22	0·02	0·19	0·01	0·19	0·03	0·19	0·02	0·15	0·02			–4·3	0·001
	40–59 years	0·43	0·02	0·38	0·02	0·35	0·02	0·33	0·02	0·34	0·02	0·29	0·01	0·23	0·02	0·26	0·02	0·25	0·02	0·24	0·01	0·23	0·02	0·25	0·02	0·25	0·02			–5·6	< 0·001
	≥ 60 years	0·49	0·02	0·52	0·02	0·52	0·02	0·46	0·02	0·47	0·02	0·44	0·01	0·37	0·02	0·35	0·02	0·36	0·02	0·35	0·01	0·32	0·02	0·35	0·02	0·31	0·02			–4·4	< 0·001
Pickled vegetables	20–39 years	0·24	0·02	0·21	0·02	0·23	0·02	0·23	0·02	0·20	0·02	0·17	0·01	0·14	0·01	0·14	0·02	0·18	0·02	0·16	0·01	0·15	0·02	0·14	0·02	0·12	0·12	2007	2010	0	0·994
																												2010	2013	–14·5	0·204
																												2013	2016	4·6	0·762
																												2016	2019	–9·1	0·340
	40–59 years	0·40	0·02	0·35	0·02	0·32	0·02	0·29	0·02	0·29	0·02	0·26	0·01	0·19	0·01	0·23	0·02	0·20	0·02	0·20	0·01	0·18	0·01	0·22	0·02	0·18	0·02			–6·8	< 0·001
	≥ 60 years	0·45	0·02	0·48	0·02	0·46	0·02	0·42	0·02	0·43	0·02	0·40	0·01	0·33	0·02	0·31	0·02	0·32	0·02	0·32	0·01	0·28	0·02	0·32	0·02	0·27	0·02			–4·6	< 0·001
Unprocessed fish and shellfish	20–39 years	0·08	0·01	0·06	0·01	0·06	0	0·06	0	0·09	0·02	0·06	0	0·05	0	0·05	0	0·05	0	0·05	0	0·04	0	0·04	0	0·05	0·01			–4·3	< 0·001
	40–59 years	0·11	0·01	0·08	0	0·09	0	0·09	0	0·08	0	0·07	0	0·07	0	0·07	0	0·06	0·01	0·06	0	0·06	0	0·05	0	0·05	0	2007	2009	13·7	–
																												2009	2019	–5·8	< 0·001
	≥ 60 years	0·11	0	0·11	0·01	0·12	0·01	0·09	0	0·11	0	0·10	0	0·10	0	0·09	0	0·09	0	0·08	0	0·08	0	0·08	0	0·07	0			–3·3	< 0·001
Dried fish and shellfish	20–39 years	0·03	0·01	0·04	0·01	0·04	0·01	0·03	0·01	0·03	0·03	0·04	0·01	0·04	0·01	0·03	0·02	0·04	0·01	0·03	0·01	0·04	0·02	0·03	0·01	0·02	0·01	2007	2009	0·7	0·985
																												2009	2014	0·7	–
																												2014	2019	–14·0	–
	40–59 years	0·04	0·01	0·05	0·01	0·05	0·01	0·03	0·01	0·04	0·01	0·04	0	0·04	0·01	0·03	0·01	0·06	0·01	0·04	0	0·05	0·01	0·04	0·01	0·04	0·01			0	0·988
	≥ 60 years	0·06	0·01	0·06	0·01	0·05	0·01	0·06	0·01	0·05	0·01	0·05	0	0·04	0·01	0·04	0·01	0·04	0·01	0·04	0	0·05	0·01	0·04	0·01	0·03	0·01			–5·4	< 0·001
Processed fish and shellfish	20–39 years	0·13	0·01	0·13	0·01	0·16	0·01	0·13	0·01	0·15	0·03	0·14	0·01	0·14	0·01	0·14	0·02	0·09	0·01	0·13	0·01	0·12	0·02	0·12	0·01	0·11	0·01			–1·8	0·060
	40–59 years	0·19	0·01	0·16	0·01	0·17	0·01	0·16	0·01	0·17	0·01	0·18	0·01	0·16	0·01	0·17	0·01	0·15	0·01	0·14	0·01	0·15	0·01	0·13	0·01	0·13	0·01			–2·4	0·001
	≥ 60 years	0·20	0·01	0·19	0·01	0·20	0·01	0·21	0·01	0·21	0·01	0·21	0·01	0·22	0·01	0·19	0·01	0·17	0·01	0·19	0·01	0·19	0·01	0·20	0·01	0·17	0·01			–0·9	0·135
Unprocessed meats	20–39 years	0·04	0	0·03	0	0·04	0	0·04	0	0·04	0	0·04	0	0·05	0	0·05	0	0·04	0	0·05	0	0·06	0·01	0·09	0·01	0·08	0·01			3·4	0·008
	40–59 years	0·03	0	0·03	0	0·03	0	0·04	0	0·03	0	0·04	0	0·04	0	0·04	0	0·04	0	0·04	0	0·05	0	0·06	0	0·08	0	2007	2016	3·1	0·046
																												2016	2019	23·7	0·001
	≥ 60 years	0·02	0	0·02	0	0·02	0	0·02	0	0·02	0	0·02	0	0·03	0	0·03	0	0·03	0	0·03	0	0·03	0	0·05	0	0·05	0			9·6	< 0·001
Processed meats	20–39 years	0·17	0·01	0·12	0·01	0·19	0·01	0·18	0·01	0·18	0·01	0·16	0·01	0·15	0·01	0·18	0·01	0·16	0·01	0·18	0·01	0·17	0·01	0·20	0·01	0·22	0·02			1·2	0·200
	40–59 years	0·14	0·01	0·12	0·01	0·14	0·01	0·15	0·01	0·16	0·01	0·16	0	0·16	0·01	0·17	0·01	0·15	0·01	0·16	0·01	0·17	0·01	0·16	0·01	0·16	0·01			0	–
	≥ 60 years	0·09	0·01	0·09	0·01	0·10	0·01	0·10	0·01	0·11	0·01	0·11	0	0·12	0·01	0·13	0·01	0·12	0·01	0·12	0	0·13	0·01	0·13	0·01	0·15	0·01			2·2	< 0·001

NHNS, National Health and Nutrition Survey; APC, annual percentage change; SE, standard error.

* The *P*-value was analysed by the Joinpoint regression analysis.

**Table 4.** Trends in salt intake from food groups except for seasonings according to age group in Japanese women based on 2007–2019 NHNS data (Mean values and standard errors)

		2007		2008		2009		2010		2011		2012		2013		2014		2015		2016		2017		2018		2019		Lower end point	Upper end point	APC (%)	*P* *
*n*	20–39 years	764		701		692		629		602		2012		539		477		409		1398		325		384		288					
	40–59 years	1114		1110		1094		996		893		3464		889		904		931		2807		793		809		675					
	≥ 60 years	1439		1673		1604		1509		1451		5709		1545		1545		1400		5066		1335		1329		1148					
g/1000 kcal		Mean	se	Mean	se	Mean	se	Mean	se	Mean	se	Mean	se	Mean	se	Mean	se	Mean	se	Mean	se	Mean	se	Mean	se	Mean	se				
Grains	20–39 years	0·60	0·03	0·54	0·03	0·57	0·03	0·63	0·03	0·62	0·03	0·55	0·02	0·63	0·03	0·54	0·03	0·57	0·04	0·53	0·02	0·56	0·04	0·67	0·04	0·62	0·05			–0·1	0·893
	40–59 years	0·52	0·02	0·46	0·02	0·53	0·02	0·54	0·02	0·61	0·03	0·52	0·01	0·56	0·02	0·54	0·02	0·51	0·02	0·54	0·01	0·55	0·02	0·58	0·02	0·58	0·03			0·8	0·069
	≥ 60 years	0·42	0·02	0·43	0·01	0·41	0·01	0·47	0·02	0·45	0·02	0·42	0·01	0·48	0·01	0·47	0·02	0·44	0·01	0·45	0·01	0·48	0·01	0·47	0·01	0·52	0·02			1·2	0·008
Bread	20–39 years	0·27	0·01	0·24	0·01	0·26	0·01	0·29	0·02	0·29	0·01	0·25	0·01	0·28	0·02	0·28	0·02	0·28	0·02	0·24	0·01	0·25	0·02	0·24	0·02	0·24	0·02			–0·6	0·376
	40–59 years	0·27	0·01	0·22	0·01	0·27	0·01	0·29	0·01	0·28	0·01	0·27	0·01	0·30	0·01	0·28	0·01	0·29	0·01	0·28	0·01	0·29	0·01	0·28	0·01	0·26	0·01			0·5	0·330
	≥ 60 years	0·22	0·01	0·23	0·01	0·24	0·01	0·25	0·01	0·26	0·01	0·23	0	0·28	0·01	0·26	0·01	0·27	0·01	0·26	0	0·28	0·01	0·25	0·01	0·27	0·01			3·1	< 0·001
Noodles	20–39 years	0·11	0·01	0·09	0·01	0·12	0·01	0·12	0·02	0·13	0·01	0·11	0	0·14	0·02	0·11	0·02	0·11	0·01	0·12	0·01	0·11	0·01	0·20	0·03	0·21	0·03	2007	2012	0·0	–
																												2012	2016	0·9	0·900
																												2016	2019	20·0	0·133
	40–59 years	0·10	0·01	0·08	0·01	0·10	0·01	0·09	0·01	0·11	0·01	0·10	0	0·10	0·01	0·09	0·01	0·09	0·01	0·09	0	0·09	0·01	0·15	0·01	0·14	0·01	2007	2016	–2·6	–
																												2016	2019	19·5	0·067
	≥ 60 years	0·22	0·01	0·23	0·01	0·23	0·01	0·24	0·01	0·25	0·01	0·23	0	0·28	0·01	0·25	0·01	0·26	0·01	0·25	0	0·27	0·01	0·25	0·01	0·27	0·01			2·1	< 0·001
Instant noodles	20–39 years	0·15	0·03	0·14	0·02	0·13	0·02	0·17	0·03	0·16	0·03	0·14	0·01	0·14	0·03	0·10	0·02	0·12	0·03	0·12	0·01	0·14	0·03	0·15	0·04	0·13	0·03			–1·8	0·071
	40–59 years	0·10	0·02	0·10	0·02	0·12	0·02	0·12	0·02	0·19	0·03	0·11	0·01	0·10	0·02	0·12	0·02	0·07	0·01	0·10	0·01	0·11	0·02	0·09	0·02	0·12	0·02			–1·8	0·360
	≥ 60 years	0·08	0·01	0·06	0·01	0·05	0·01	0·11	0·01	0·07	0·01	0·08	0·01	0·07	0·01	0·08	0·01	0·06	0·01	0·07	0·01	0·07	0·01	0·07	0·01	0·10	0·02			–0·9	0·598
Vegetables	20–39 years	0·27	0·02	0·25	0·02	0·23	0·02	0·23	0·02	0·27	0·02	0·20	0·01	0·60	0·43	0·20	0·02	0·22	0·03	0·19	0·01	0·17	0·02	0·19	0·03	0·17	0·02			–3·6	< 0·001
	40–59 years	0·39	0·02	0·41	0·02	0·32	0·02	0·28	0·02	0·30	0·02	0·28	0·01	0·27	0·02	0·22	0·01	0·24	0·02	0·25	0·01	0·20	0·01	0·20	0·01	0·19	0·02			–5·9	< 0·001
	≥ 60 years	0·55	0·02	0·55	0·02	0·49	0·02	0·46	0·02	0·49	0·02	0·45	0·01	0·40	0·02	0·40	0·02	0·38	0·02	0·37	0·01	0·31	0·02	0·35	0·02	0·31	0·02			–4·6	< 0·001
Pickled vegetables	20–39 years	0·23	0·02	0·20	0·02	0·19	0·02	0·18	0·02	0·22	0·02	0·16	0·01	0·56	0·43	0·16	0·02	0·17	0·03	0·15	0·01	0·13	0·02	0·16	0·03	0·13	0·02			–4·1	< 0·001
	40–59 years	0·34	0·02	0·36	0·02	0·28	0·02	0·24	0·02	0·26	0·02	0·23	0·01	0·23	0·02	0·17	0·01	0·19	0·02	0·21	0·01	0·16	0·01	0·16	0·01	0·16	0·02			–6·7	< 0·001
	≥ 60 years	0·51	0·02	0·49	0·02	0·44	0·02	0·41	0·02	0·44	0·02	0·40	0·01	0·35	0·02	0·35	0·02	0·33	0·02	0·33	0·01	0·27	0·02	0·31	0·02	0·27	0·02			–4·9	< 0·001
Unprocessed fish and shellfish	20–39 years	0·07	0	0·06	0	0·06	0	0·08	0·01	0·08	0·01	0·06	0	0·06	0	0·06	0	0·06	0·01	0·05	0	0·04	0·01	0·05	0	0·05	0			–2·3	< 0·001
	40–59 years	0·10	0·01	0·09	0·01	0·07	0	0·08	0	0·07	0	0·07	0	0·07	0	0·07	0	0·07	0	0·06	0	0·05	0	0·05	0	0·05	0			–3·9	< 0·001
	≥ 60 years	0·11	0	0·09	0	0·10	0	0·09	0	0·10	0	0·09	0	0·09	0	0·09	0	0·09	0	0·08	0	0·08	0	0·07	0	0·07	0			–2·9	< 0·001
Dried fish and shellfish	20–39 years	0·04	0·01	0·05	0·01	0·05	0·01	0·03	0·01	0·04	0·01	0·04	0	0·04	0·01	0·03	0·01	0·06	0·01	0·04	0	0·05	0·01	0·04	0·01	0·04	0·01	2007	2013	–2·8	0·236
																												2013	2016	0·9	0·972
																												2016	2019	2·4	0·833
	40–59 years	0·06	0·01	0·07	0·01	0·06	0·01	0·06	0·01	0·06	0·01	0·06	0	0·06	0·01	0·04	0·01	0·04	0·01	0·05	0	0·05	0·01	0·04	0·01	0·03	0·01			–4·5	< 0·001
	≥ 60 years	0·11	0·01	0·11	0·01	0·10	0·01	0·11	0·01	0·10	0·01	0·10	0	0·11	0·01	0·09	0·01	0·11	0·01	0·09	0	0·09	0·01	0·09	0·01	0·08	0·01			–2·6	< 0·001
Processed fish and shellfish	20–39 years	0·14	0·01	0·13	0·01	0·15	0·01	0·13	0·01	0·17	0·02	0·14	0·01	0·16	0·02	0·16	0·02	0·13	0·02	0·14	0·01	0·14	0·02	0·10	0·01	0·12	0·02			–1·0	0·260
	40–59 years	0·19	0·01	0·17	0·01	0·17	0·01	0·18	0·01	0·16	0·01	0·18	0·01	0·19	0·01	0·17	0·01	0·14	0·01	0·16	0·01	0·15	0·01	0·15	0·01	0·13	0·01			–2·0	0·006
	≥ 60 years	0·20	0·01	0·19	0·01	0·19	0·01	0·20	0·01	0·22	0·01	0·20	0·01	0·21	0·01	0·19	0·01	0·18	0·01	0·19	0·01	0·19	0·01	0·20	0·01	0·18	0·01			–0·5	0·239
Unprocessed meats	20–39 years	0·03	0	0·03	0	0·04	0	0·04	0	0·04	0	0·04	0	0·04	0	0·04	0	0·04	0	0·05	0	0·04	0	0·08	0·01	0·08	0·01			3·0	0·008
	40–59 years	0·03	0	0·03	0	0·03	0	0·03	0	0·03	0	0·03	0	0·04	0	0·04	0	0·04	0	0·04	0	0·04	0	0·06	0	0·07	0	2007	2017	4·5	0·001
																												2017	2019	28·9	0·003
	≥ 60 years	0·02	0	0·02	0	0·02	0	0·02	0	0·02	0	0·02	0	0·02	0	0·03	0	0·03	0	0·03	0	0·03	0	0·05	0	0·05	0			10·0	< 0·001
Processed meats	20–39 years	0·18	0·01	0·13	0·01	0·18	0·01	0·16	0·01	0·18	0·01	0·17	0·01	0·18	0·01	0·18	0·01	0·16	0·01	0·17	0·01	0·14	0·01	0·19	0·01	0·19	0·02			0·4	0·671
	40–59 years	0·14	0·01	0·11	0·01	0·16	0·01	0·14	0·01	0·17	0·01	0·16	0	0·16	0·01	0·16	0·01	0·15	0·01	0·15	0	0·16	0·01	0·16	0·01	0·19	0·01			–1·6	< 0·001
	≥ 60 years	0·09	0·01	0·09	0	0·10	0	0·10	0·01	0·10	0·01	0·11	0	0·12	0·01	0·12	0·01	0·11	0·01	0·12	0	0·12	0·01	0·12	0·01	0·13	0·01	2007	2009	11·1	0·121
																												2009	2013	3·2	0·221
																												2013	2019	1·9	0·107

NHNS, National Health and Nutrition Survey; APC, annual percentage change; SE, standard error.

* The *P*-value was analysed by the Joinpoint regression analysis.

**Fig. 2.**
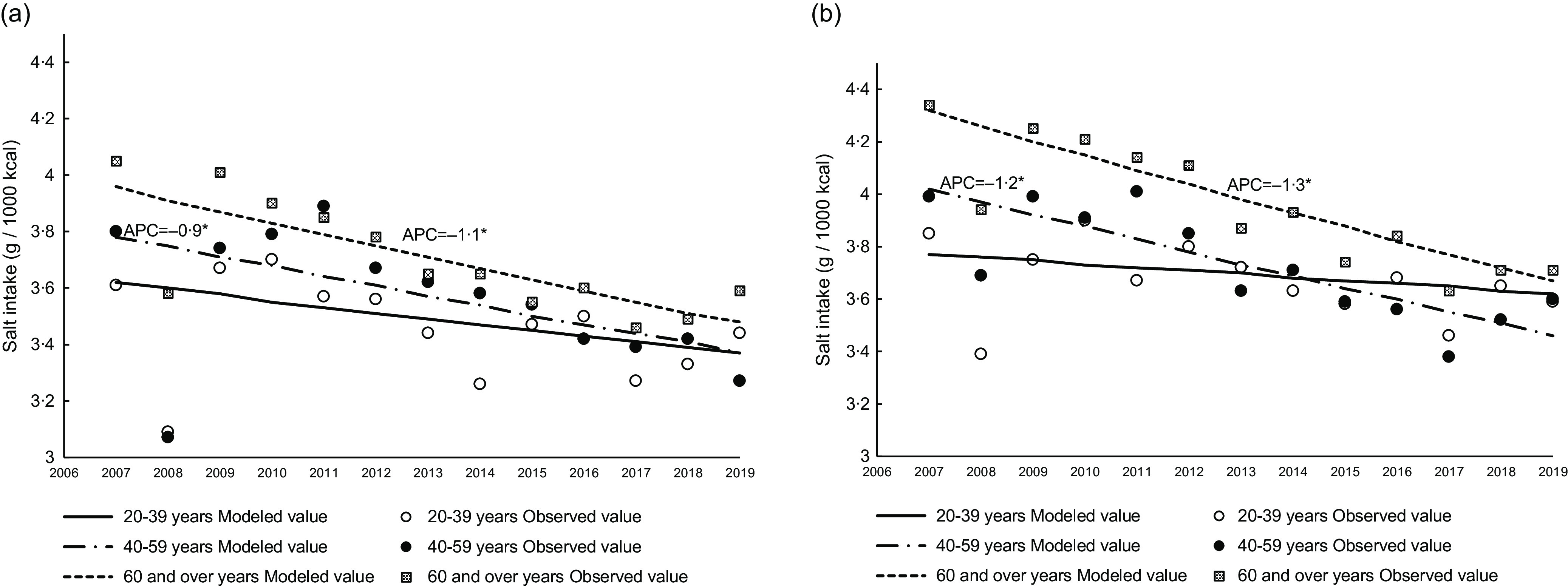
Trends in salt intake from seasonings according to age group in Japanese men (a) and women (b) based on 2007–2019 NHNS data. Trends in salt intake were analysed by the Joinpoint regression analysis (*P* < 0·05). APC, annual percentage change; NHNS, National Health and Nutrition Survey.

**Fig. 3.**
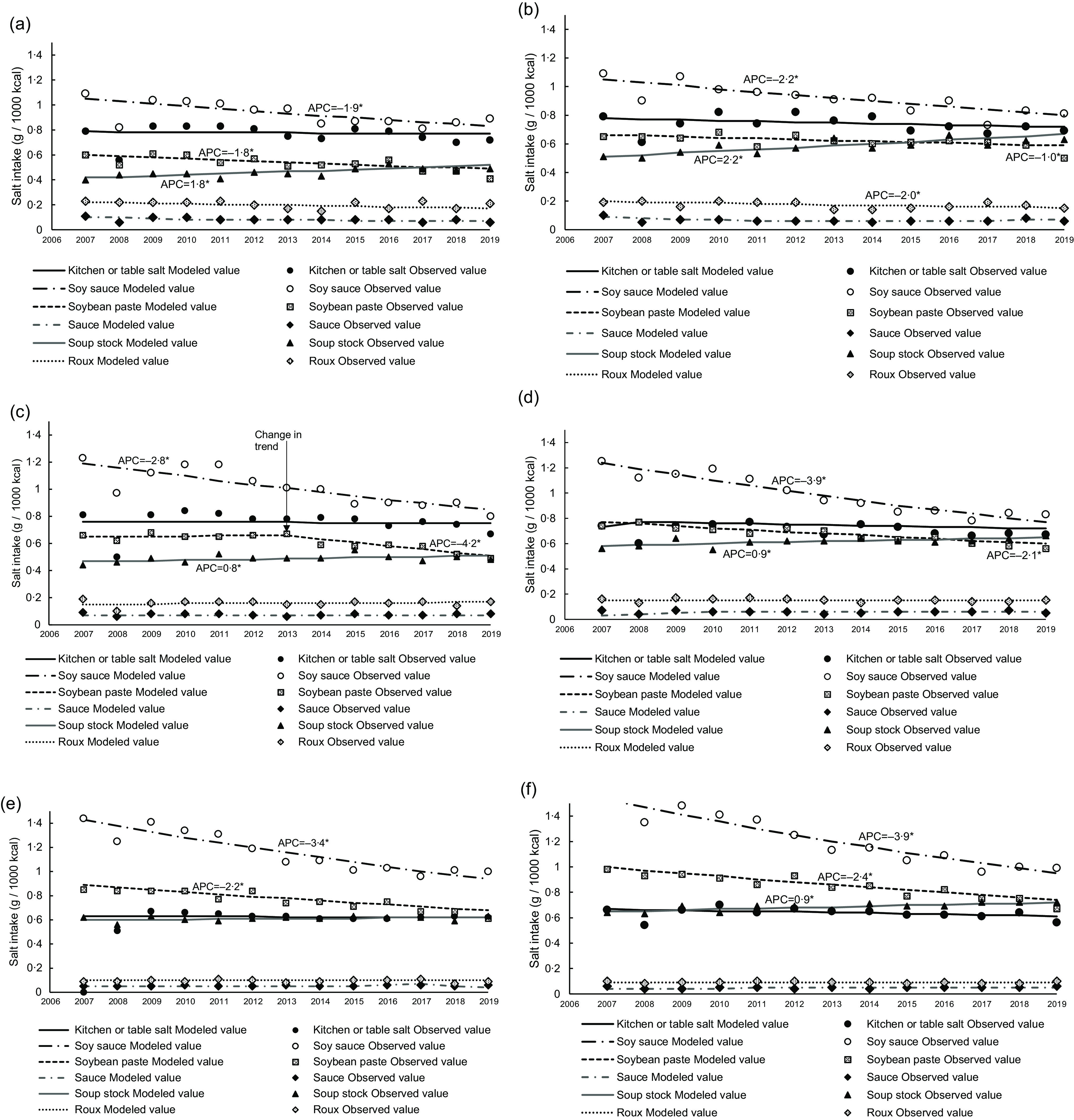
Trends in salt intake from seasonings that contribute particularly to salt intake in men aged 20–39 years (a), women aged 20–39 years (b), men aged 40–59 years (c), women aged 40–59 years (d), men aged ≥ 60 years (e) and women aged ≥ 60 years (f) based on 2007–2019 NHNS data. Trends in salt intake were analysed by the Joinpoint regression analysis (*P* < 0·05). APC, annual percentage change; NHNS, National Health and Nutrition Survey.

The trends in salt intake from seasonings between 2007 and 2019 are shown in Fig. 2(a) and (b). Salt intake from seasonings in both men and women, except for those aged 20–39 years, decreased over time with an APC of −0·9 % (men aged 40–59 years), −1·1 % (men aged ≥ 60 years), −1·2 % (women aged 40–59 years) and −1·3 % (women aged ≥ 60 years). Fig. 3(a)–(f) shows the trend in six major seasonings that contribute to salt intake (i.e. seasonings that have continuously been a source of excess salt intake at approximately 0·05 g/1000 kcal between 2007 and 2019). Supplementary Tables 2 and 3 show the trends in salt intake from various seasonings. Salt intake from soya sauce and soyabean paste decreased over time (between 2013 and 2019 for only soyabean paste in men aged 40–59 years), with the APC decreasing from −1·0 % to −4·2 %. Additionally, salt intake from soup stock in all age categories (except for men aged ≥ 60 years) showed a significant increase. Moreover, salt intake from dressings in men aged < 60 years significantly increased. Salt intake from other seasonings increased in men aged 20–39 years and in women aged 40–59 years, with an APC of 59·6 % (2017–2019) and 28·6 % (2016–2019), respectively.

## Discussion

This study described the trends in dietary salt sources based on the 2007–2019 NHNS data. To our knowledge, this is the first study to investigate the trends in dietary salt sources among Japanese adults. We found that salt intake decreased over time between 2007 and 2019 and the main source of dietary Na was seasonings. Salt intake from seasonings was unchanged in both men and women aged 20–39 years, despite a slight decrease in men and women aged 40–59 and ≥ 60 years. Salt intake from soya sauce and soyabean paste decreased over time across all age categories for both men and women.

Salt intake (g/1000 kcal) decreased over time. However, salt intake in men aged ≥ 60 years has not changed since 2017. One reason for this stable salt intake in men aged ≥ 60 years is that the decrease in the consumption of Na dietary sources (i.e. pickled vegetable, unprocessed fish and shellfish, and dried fish and shellfish) was not sufficient to compensate for the increase in other foods (i.e. bread) in men compared with women, particularly in those aged ≥ 60 years, as shown in Supplementary Tables 4 and 5. As a result, the annual decrease in the percentage of salt intake from pickled vegetables, unprocessed fish and shellfish, and dried fish and shellfish was higher among women aged ≥ 60 years than among men. Additionally, the increase in salt intake from processed meat in men aged ≥ 60 years (not women) may explain the difference in the trends in salt intake between men and women. Although sex differences were not considered, a previous study of Japanese adults also reported an increase in the ‘bread and dairy’ and ‘animal food and oil’ dietary patterns, especially among people aged ≥ 50 years, and a decrease in the ‘plant food and fish’ pattern^(31)^. As dietary patterns become more westernised, the food sources of salt may change further; thus, it will be necessary to assess these trends in dietary salt sources.

Although the salt intake (g/1000 kcal) of those aged 20–39 years was lower than that of those aged 40–59 years in 2007, the decrease in salt intake was the lowest among men and women aged 20–39 years; thus, the salt intake (g/1000 kcal) of those aged 20–39 years was the same or higher than that of those aged 40–59 years in 2019. This may be partly due to salt intake from seasonings, which are the largest contributor to Na consumption (approximately 70 % of total intake for all age groups), as reported in previous studies^(16,17)^. However, we found that salt intake from seasonings did not decrease over time in both men and women aged 20–39 years, unlike in those aged ≥ 40 years. As mentioned above, the westernised dietary patterns may have led to a lower salt intake than East Asian dietary patterns^(8)^. This change was more evident in older individuals than in younger ones^(31)^, which may explain the age variation in salt intake. Additionally, changing the food environment has a greater impact on improving salt reduction than changing consumer choices^(32)^. In 2013, the Japanese Society of Hypertension Salt Reduction Committee began to certify products with lower salt content, and as of 2019, more than 200 products have been certified^(33)^. However, as seasonings still remain the main source of salt intake, it may be necessary to target seasonings for population-based strategies. Reformulation is the most effective salt reduction strategy reported in developed countries such as UK and USA, where processed foods are the main source of salt intake^(34–36)^. However, unlike these Western countries, the main source of salt intake in Japan is seasonings (discretionary salt). Therefore, in addition to reformulation including the adherence to the salt intake standards proposed by the WHO^(37)^, Japan may also require strategies such as increasing the availability of salt substitutes (e.g. potassium salt), reducing their cost and consumer education^(36,38)^. Further studies investigating how seasonings (e.g. eating occasions and location of food preparation) are consumed can provide more appropriate insight into effective salt reduction strategies for the Japanese population.

Focusing on salt intake from seasonings, salt intake from soya sauce and soyabean paste decreased in men and women of all age groups. Meanwhile, except for men aged ≥ 60 years, salt intake from soup stock increased over time, although the rate of increase was lower than the rate of decrease of soya sauce or soyabean paste. Increasing umami flavour of soup stock is suggested as a method to reduce salt intake, requiring less added salt, soya sauce and soyabean paste, while maintaining salty preference^(39–41)^, and this is disseminated to the public as useful information^(42)^. These may explain our results, that is, the decrease of salt intake from soya sauce or soyabean paste and the increase of salt intake from soup stock. Moreover, reduced-salt soya sauce, soyabean paste and soup stock are becoming more commonly available. However, salt intake from these food products cannot be accurately assessed due to the lack of information on reduced-salt soya sauce and soyabean paste in the Standard Tables of Food Composition in Japan until 2017, still with the lack of reduced-salt soup stock^(28)^. Therefore, our study cannot form definitive conclusions on the changes in salt intake owing to the reduced-salt products. In the future, it will be necessary to observe changes in salt intake from soya sauce and soyabean paste, including salt-reduced products. The reduction in salt intake from soya sauce and soyabean paste could also be due to the inclusion of more food items in the seasoning group along with the revision of the Standard Table of Food Composition in Japan. Some of these seasonings were coded as soya sauce or soyabean paste prior to the revision.

Salt intake from dressings has also been reported to increase over time in the USA^(18)^. Similarly, salt intake from dressings increased in men aged < 60 years, although this trend was not observed in women. This discrepancy may be explained by the higher frequency of eating out in men than in women^(43)^ because dressings are seasonings with a high Na content among the foods commonly consumed in restaurants^(44)^. Furthermore, because of their small contribution to salt intake and the high salt content of dressings, this discrepancy is partly due to the influence of the revision in the Standard Table of Food Composition in Japan^(27,28)^. However, the revision of food composition databases is inevitable and even necessary to observe the trends in dietary intake by capturing the changes in the types of foods consumed. Therefore, it is necessary to consider the effect of the revision of food composition databases on the trend of dietary intake in future surveys.

This study had several strengths. First, we used the NHNS data, the only nationally representative survey data available on the dietary intake among Japanese. Additionally, as NHNS is conducted annually, assessing dietary trends at the population level is possible. However, several limitations need to be addressed. First, the participants were randomly selected from nationally representative households in Japan in the NHNS; however, response rates, ranging from 44·4 % in 2016 to 68·6 % in 2010, varied widely and are considered relatively low, and the individual-level response rate is unknown. These factors might have introduced some bias in the estimation of the average salt intake. Second, data from participants with missing weight and height values were excluded from the analysis. Those excluded had a significantly lower energy intake. Additionally, those excluded aged under 60 years were younger and those excluded aged 60 years and older than the participants in this study, respectively (data not shown). This may have affected the results of this study. However, Japanese adults who did not participate in physical examinations (missing height and weight data) have been reported to underreport their energy intake^(45)^. Therefore, results from our study are likely to provide a more accurate assessment than results which include participants with no height or weight data. Third, the validity of the estimation of the individual intake of energy and nutrients based on household-based dietary records compared with those on self-reported data has been examined only among young women^(46)^. Thus, the utility of this method applied to other age or sex categories remains unknown. Fourth, the nutrient values of the Standard Table of Food Composition in Japan are presented as average values of different products; thus, there could be variations in the salt content of commercial foods that may cause an underestimation or overestimation of salt intake. However, variations in nutrient composition (not only for salt but also for other nutrients) are a general limitation of studies based on dietary surveys. Fifth, although the onset of chronic disease is one of the possible causes of changes in dietary habits, we could not consider it in the present study due to a lack of participant medical histories in the NHNS. However, the NHNS excludes those with unusual dietary habits, such as exclusive consumption of liquid foods or medication due to illness or other reasons. Thus, the onset of chronic disease would not substantially influence the present findings.

This study identified a decrease in salt intake over time and that salt intake from seasonings decreased in those aged ≥ 40 years; however, this was not observed in those aged 20–39 years. Additionally, seasonings remain as the main dietary Na source across all age categories. Hence, it may be necessary to focus on seasonings to establish salt reduction guidelines in Japan. Further studies on the many types of seasonings, including occasional consumption and timing, are required. Continued monitoring of trends in dietary salt sources is also needed.

## Supplementary material

For supplementary material accompanying this paper visit https://doi.org/10.1017/S0007114522001416. click here to view supplementary material
